# Potential effectiveness of a therapeutic HPV intervention campaign in Uganda

**DOI:** 10.1002/ijc.33867

**Published:** 2021-11-24

**Authors:** Jennifer C. Spencer, Nicole G. Campos, Emily A. Burger, Stephen Sy, Jane J. Kim

**Affiliations:** ^1^ Department of Population Health Dell Medical School, University of Texas at Austin Austin Texas USA; ^2^ Department of Internal Medicine Dell Medical School, University of Texas at Austin Austin Texas USA; ^3^ Center for Health Decision Science Harvard T.H. Chan School of Public Health Boston Massachusetts USA; ^4^ Department of Health Management and Health Economics University of Oslo Oslo Norway

**Keywords:** cervical cancer, human papillomavirus, prevention, simulation modeling, vaccine

## Abstract

Cervical cancer is a major source of morbidity and mortality in Uganda. In addition to prophylactic HPV vaccination, secondary prevention strategies are needed to reduce cancer burden. We evaluated the potential cancer reductions associated with a hypothetical single‐contact therapeutic HPV intervention—with 70% coverage and variable efficacy [30%‐100%]—using a three‐stage HPV modeling framework reflecting HPV and cervical cancer burden in Uganda. In the reference case, we assumed prophylactic preadolescent HPV vaccination starting in 2020 with 70% coverage. A one‐time therapeutic intervention targeting 35‐year‐old women in 2025 (not age‐eligible for prophylactic vaccination) averted 1801 cervical cancers per 100 000 women over their lifetime (100% efficacy) or 533 cancers per 100 000 (30% efficacy). Benefits were considerably smaller in birth cohorts eligible for prophylactic HPV vaccination (768 cases averted per 100 000 at 100% efficacy). Evaluating the population‐level impact over 40 years, we found introduction of a therapeutic intervention in 2025 with 100% efficacy targeted annually to 30‐year‐old women averted 139 000 incident cervical cancers in Uganda. This benefit was greatly reduced if efficacy was lower (30% efficacy; 41 000 cases averted), introduction was delayed (2040 introduction; 72 000 cases averted) or both (22 000 cases averted). We demonstrate the potential benefits of a single‐contact HPV therapeutic intervention in a low‐income setting, but show the importance of high therapeutic efficacy and early introduction timing relative to existing prophylactic programs. Reduced benefits from a less efficacious intervention may be somewhat offset if available within a shorter time frame.

## Graphical abstract

What's new?

The authors simulated the potential cervical cancer reduction associated with a hypothetical, single‐contact human papillomavirus (HPV) therapeutic intervention under different intervention and delivery scenarios in Uganda. With perfect efficacy against HPV 16/18, an intervention with 70% coverage could avert 139 000 cancers over 40 years. The estimated benefit was highest at ages 30‐35 and for cohorts without high prophylactic HPV vaccine coverage. Reduced benefits from a less efficacious intervention could be partly offset if the intervention became available within a shorter time frame. This exploratory analysis could help inform priority setting around cervical cancer prevention strategies in low‐ and middle‐income countries.

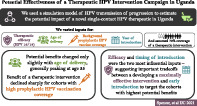

**Abbreviations**
CIN: cervical intraepithelial neoplasm
HPV: human papillomavirus
LMIC: low‐income and middle‐income countries
VIA: visual inspection with acetic acid

## 1 INTRODUCTION

Cervical cancer resulting from persistent infection with human papillomavirus (HPV) is a leading cause of cancer death worldwide, with the highest burden in low‐income and middle‐income countries (LMIC).^1^ Uganda is among the countries with the highest burden, with more than 6000 incident cervical cancer cases and 4000 cervical cancer deaths each year.^2^ Prophylactic HPV vaccination offers unprecedented potential to reduce the burden of cervical cancer in LMIC, but uptake varies widely across settings, and even in settings with high uptake, it will be decades before the impact is fully realized.^3^ The Ugandan Ministry of Health started a national prophylactic HPV vaccination program in 2015, but remains behind the national goal of 80% vaccine coverage, with estimates suggesting 20% to 50% of age‐eligible girls have received at least one dose of HPV vaccine.^4^, ^5^, ^6^ Other low income countries with similarly high rates of cervical cancer have not yet started HPV vaccination programs, including Burundi, Malawi and Madagascar.^7^ To reach targets for global elimination of cervical cancer as a public health problem, LMIC must both improve HPV vaccine uptake and scale up secondary prevention practices.^3^, ^8^

Cervical cancer screening remains underutilized in LMIC as many traditional approaches to screening and treatment of precancerous disease present challenges where resources, personnel and health budgets are limited.^9^, ^10^ Given existing barriers to accessing care, strategies that reduce the number of visits needed for diagnosis and treatment are generally cost‐effective relative to traditional cytology approaches in which multiple follow‐up visits are needed.^11^, ^12^ In Uganda, lifetime screen rates are estimated to be between 5% and 30% and typically relies on visual inspection with acetic acid (VIA) followed by treatment with cryotherapy for women with identified precancerous lesions.^10^, ^13^ New developments in delivery of cervical cancer screening seek to reduce costs and increase accessibility to treatment by reducing complexity and resource burden of traditional screening strategies.^14^, ^15^, ^16^

One potential approach to reducing cervical cancer burden is the development of an HPV therapeutic intervention. This approach would use a therapeutic intervention (potentially a postexposure vaccine, vaginal suppository or antiviral medication) for treating an active HPV infection which could be delivered noninvasively in a single visit without specialized clinical expertise. Numerous candidate therapeutics have been tested for safety or efficacy,^17^, ^18^, ^19^ but to date these have shown only small effects, with HPV clearance over 6 to 9 months around 15% to 20% higher than placebo.^20^, ^21^ To inform investment for future research in therapeutic interventions as the landscape of cervical cancer prevention continues to change, we used a simulation modeling approach of HPV transmission and carcinogenesis to assess the potential cervical cancer reduction associated with broad use of a single‐contact HPV therapeutic intervention under different intervention and delivery scenarios in Uganda.

## 2 METHODS

### 2.1 Overview

We used a three‐stage HPV modeling framework adapted to reflect sexual transmission of HPV, the natural history of HPV carcinogenesis and population dynamics in Uganda. We used these models to project cervical cancer incidence in the absence of any intervention compared to the introduction of a hypothetical single‐contact therapeutic HPV intervention over 40 or 100 years. We altered assumptions about prophylactic preadolescent HPV vaccination coverage as well as the efficacy, timing and target age for the therapeutic intervention to assess the impact on the effectiveness of a single‐contact HPV therapeutic.

### 2.2 Modeling approach

The development and calibration of our models have been previously described.^11^, ^22^, ^23^, ^24^ HPV incidence was estimated using Harvard‐HPV, an agent‐based model that reflects heterosexual partnership formation and sexual transmission of HPV, allowing for estimation of both direct and indirect (herd) effects of prophylactic HPV vaccination.^25^ The Harvard‐HPV model provided estimates of monthly incidence of new HPV infection by age, year and HPV genotype (for 9‐valent high‐risk vaccine types 16, 18, 31, 33, 45, 52 and 58) given assumed population coverage of prophylactic HPV vaccination over time.

For each alternative prophylactic HPV vaccination scenario, the estimated incidence of HPV infection by age and time was then incorporated into an individual‐based model of HPV‐induced cervical cancer natural history. This model (HPV‐CC) simulates monthly transitions through a series of discrete health states, including risk of noncancer mortality. Girls enter the model at age 9 with no HPV infection and can acquire HPV at age‐specific and type‐specific incidence rates. Once an HPV infection is acquired, it may clear spontaneously or progress to cervical intraepithelial neoplasia (CIN) Grade 2 or Grade 3. CIN states may regress spontaneously or may progress to an incident cervical cancer. Cervical cancer may be detected symptomatically or may progress to a more severe stage. Monthly transition probabilities can be a function of age, HPV genotype, duration of infection or lesion and history of prior HPV infection. Data were calibrated to reflect epidemiologic data from Uganda.^24^, ^26^ To report population‐level impact over time, this process was run in parallel for all birth cohorts who were alive for any portion of our study time horizon (40 or 100 years). We then age‐weighted and scaled to represent the full population of Uganda using United Nations projected population estimates to produce total annual cervical cancer incidence in Uganda for each scenario.^23^, ^27^, ^28^

### 2.3 Therapeutic strategies

We modeled a hypothetical therapeutic HPV intervention, starting with an optimistic reference case and varying therapeutic intervention characteristics to assess the effect on outcomes (Table 1). We assumed the intervention could be administered within a single visit without screening and could achieve 70% coverage in the target age range(s). Our target profile assumed 100% efficacy against prevalent HPV 16/18 infections, meaning a total clearance of all active HPV 16/18 infections and any associated CIN lesions. We also examined a range of therapeutic efficacies, including efficacy as low as 30%. We assumed the therapeutic intervention only clears active infections and has no prophylactic benefit against future acquisition or reacquisition of HPV, although we assume the same natural immunity against same‐type reinfection after therapeutic‐induced clearance as would be acquired through spontaneous clearance of an HPV infection. In our optimistic target profile, we modeled introduction of a therapeutic intervention as early as 2025; we considered introduction as late as 2040 in sensitivity analysis.

**TABLE 1:** Baseline and sensitivity values of model inputs

Parameter	Reference case	Sensitivity analysis
Therapeutic intervention characteristics		
Efficacy (*clearance of active HPV 16/18 infections/lesions*)	100%	30%, 60%, 80%
Population coverage	70%	–
Prophylactic benefit	None	–
Introduction year	2025	2030, 2035, 2040
Prophylactic vaccine characteristics		
Efficacy (*prevention of HPV 16/18/31/33/45/52/58 infections*)	100%	–
Population coverage	70%	0%, 90%
Target age (years)	9 (1 year catch‐up, ages 10‐14)	–
Duration of benefit	Lifetime	–
Introduction year	2020	–

### 2.4 Analysis

To isolate the potential interaction between a therapeutic intervention and prophylactic HPV vaccination, we first evaluated the lifetime benefits of a therapeutic intervention by age of administration for specific birth cohorts. We selected seven birth cohorts for initial comparison (Figure S1); two that were age‐eligible for prophylactic HPV vaccination (2006 and 2010) and five that were not (2000, 1995, 1990, 1985 and 1981). All birth cohorts could experience different levels of herd protection due to ongoing routine prophylactic HPV vaccination. For these selected birth cohorts, we estimated the reductions in lifetime cervical cancer cases per 100 000 women resulting from a one‐time administration of an HPV therapeutic in each birth cohort, varying the age at which the intervention was delivered (from 25 to 49 years of age). In our reference case, prophylactic HPV vaccination began in 2020 with 70% coverage of girls aged 9 to 14 years in the first year and 70% coverage of incoming 9‐year‐old cohorts annually thereafter (Table 1). Coverage of 70% is consistent with estimates of coverage in Uganda^7^, ^10^, ^13^ and reflects a conservative estimate of prophylactic impact, allowing for a higher potential benefit of therapeutic. However, to represent a range of possible HPV vaccine coverage levels in Uganda and across other low‐income countries, we also evaluate scenarios with no HPV vaccine coverage (0%) or coverage consistent with targets set by the World Health Organization (90%).^3^ We assumed lifetime efficacy of prophylactic vaccination, consistent with best evidence showing a sustained immune response in studies with long‐term follow up.^29^ To assess the impact of changing therapeutic efficacy, we compared findings for two of our representative birth cohorts, one age‐eligible for prophylactic vaccination (2010) and one not eligible (2000). We reestimated the total lifetime reductions in cervical cancer if a therapeutic intervention only cleared 80%, 60% or 30% of active HPV 16/18 infections.

To assess population‐level impact on total cervical cancer burden, we compared two strategies for delivering an ongoing therapeutic intervention; (a) routine delivery or (b) campaign delivery. We assumed both strategies begin with an expanded eligibility in the first year where therapeutic intervention is offered to all women aged 30 to 44 years. After the first year a *campaign* strategy would offer intervention every 5 years targeting women aged 30 to 34 years (Figure S2). Alternatively, a *routine* administration strategy would target women for therapeutic HPV intervention annually at age 30 years. We projected the cumulative cervical cancer cases averted through therapeutic intervention with each of these strategies compared to no therapeutic intervention over 40 years. In sensitivity analysis we vary intervention efficacy, start year and underlying coverage of prophylactic HPV vaccination among age‐eligible cohorts.

Finally, we extended the analytic time horizon from 40 to 100 years and assessed the impact of a therapeutic intervention on future cervical cancer incidence rate in Uganda with and without prophylactic HPV vaccination. For consistency of comparison, we age‐standardized the rate in future years to the Uganda population in 2020. We projected (a) cervical cancer incidence over 100 years with and without therapeutic intervention using our reference case assumption of 70% prophylactic HPV vaccination coverage; and (b) cervical cancer incidence over 100 years with and without therapeutic intervention in a setting with no existing prophylactic coverage.

## 3 RESULTS

**FIGURE 1:**
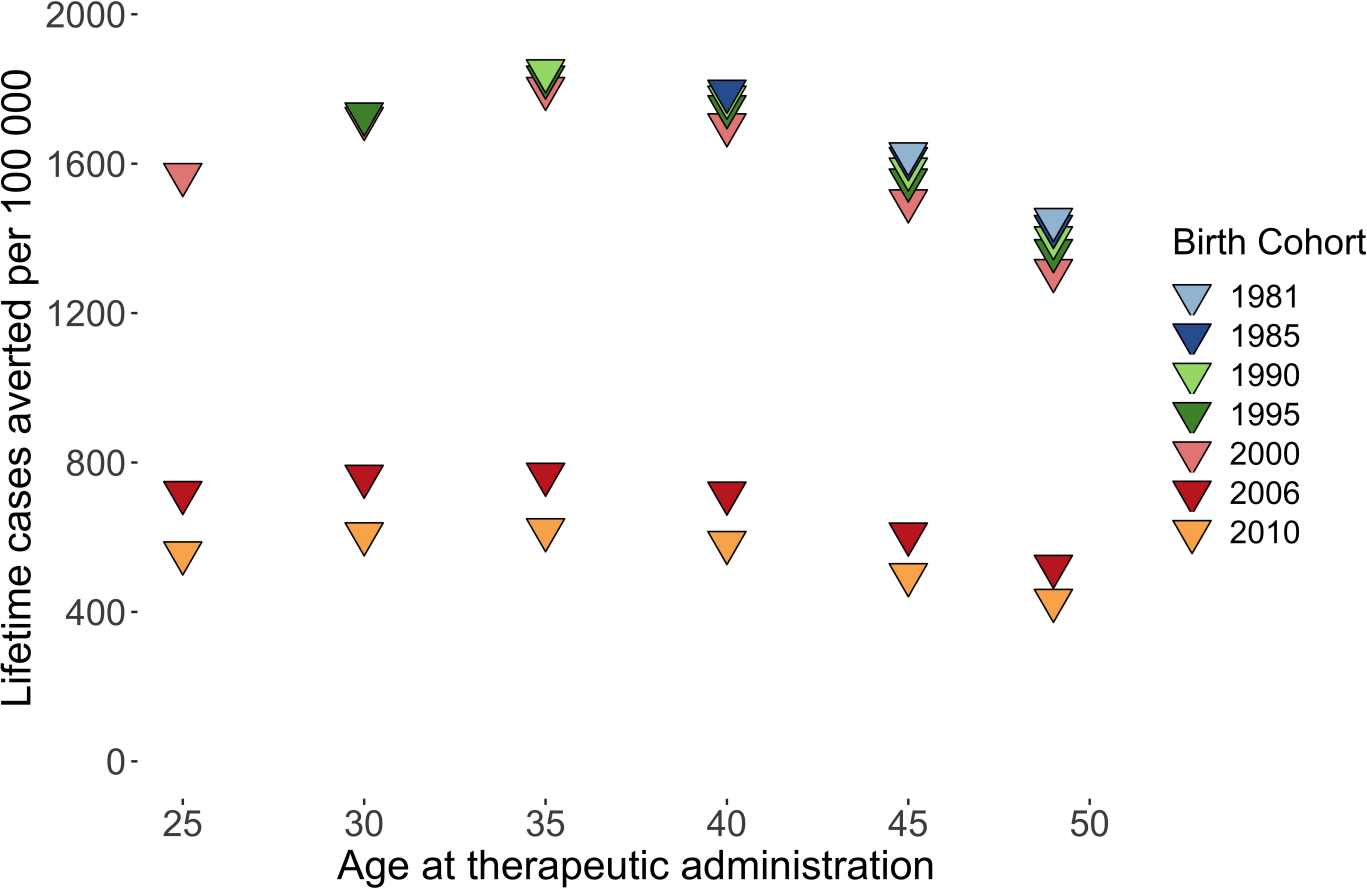
Lifetime cervical cancer cases averted through single‐contact therapeutic intervention. Demonstrates total cancer cases averted over the remaining lifetime of the birth cohort by a one‐time intervention with a theoretical HPV therapeutic targeted across ages to seven different birth cohorts. Those born in 2006 and 2010 were potentially eligible to receive prophylactic vaccination; those born earlier than this were not [Color figure can be viewed at wileyonlinelibrary.com]

The impact of an HPV therapeutic varied only moderately by age at delivery, with the highest impact at age 35 years (Figure 1). For example, in the 2000 birth cohort (which did not receive prophylactic vaccination), a therapeutic intervention averted 1801 lifetime cervical cancer cases per 100 000 women if delivered at age 35, a 31.0% relative reduction over status quo. In the same birth cohort, only 1721 cases were averted with therapeutic delivered at age 25 (a 29.6% reduction) or 1314 per 100 000 if delivered at age 49 (a 22.6% reduction). Benefits for other birth cohorts not prophylactically vaccinated were similar. However, while the relative cancer reduction remained similar, the absolute benefits of an HPV therapeutic dropped sharply for cohorts that were eligible for prophylactic vaccination (Figure 1). For those born in 2006 (70% of whom received prophylactic HPV vaccination), receipt of a therapeutic intervention at age 35 averted 768 cervical cancer cases per 100 000 (28.4% reduction), 720 cases averted per 100 000 at age 25 (26.6% reduction) and 582 cases per 100 000 at age 49 (19.2% reduction).

**FIGURE 2:**
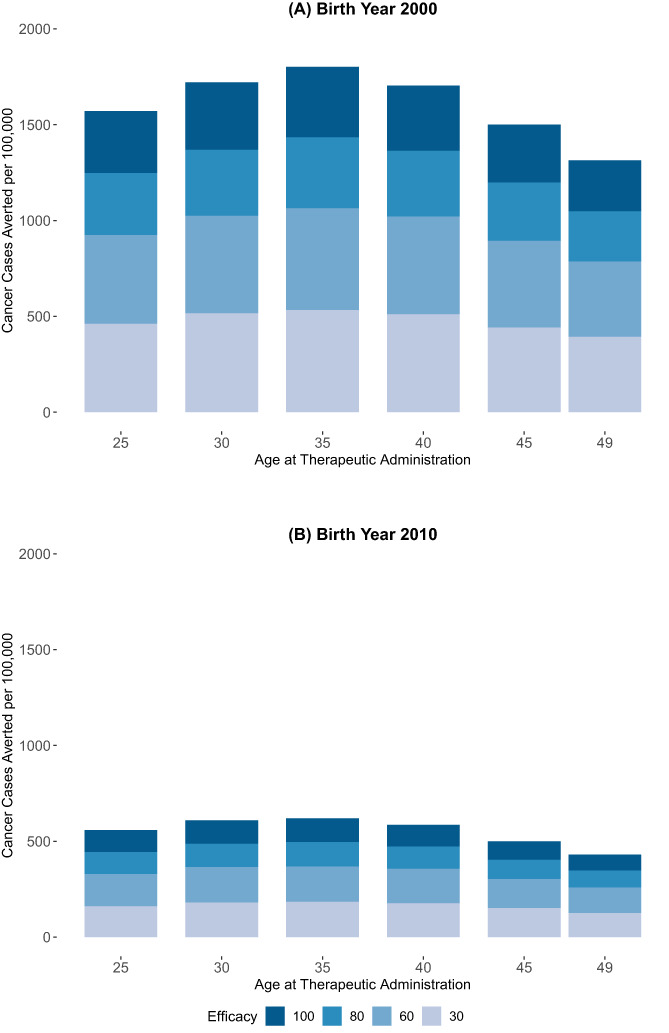
Lifetime cervical cancer cases averted through single‐contact therapeutic intervention, by efficacy and age at administration. Demonstrates total cancer cases averted over the remaining lifetime of the birth cohort by a one‐time intervention with a theoretical HPV therapeutic targeted to (A) women born in 2000 (not offered prophylactic vaccination) or (B) women born in 2010 (70% coverage of prophylactic vaccination) [Color figure can be viewed at wileyonlinelibrary.com]

As the efficacy of the therapeutic intervention declined, lifetime benefits also decreased (Figure 2). In the 2000 birth cohort, a therapeutic intervention with an efficacy of 80%, 60% or 30% averted 1434; 1063 or 533 cases per 100 000, respectively. Similar declines were seen in the 2010 birth cohort, where a therapeutic intervention with 100% efficacy delivered at age 35 averted 630 cases per 100 000, compared to 496 cases at 80% efficacy, 396 at 60% efficacy or 184 at 30% efficacy.

**FIGURE 3:**
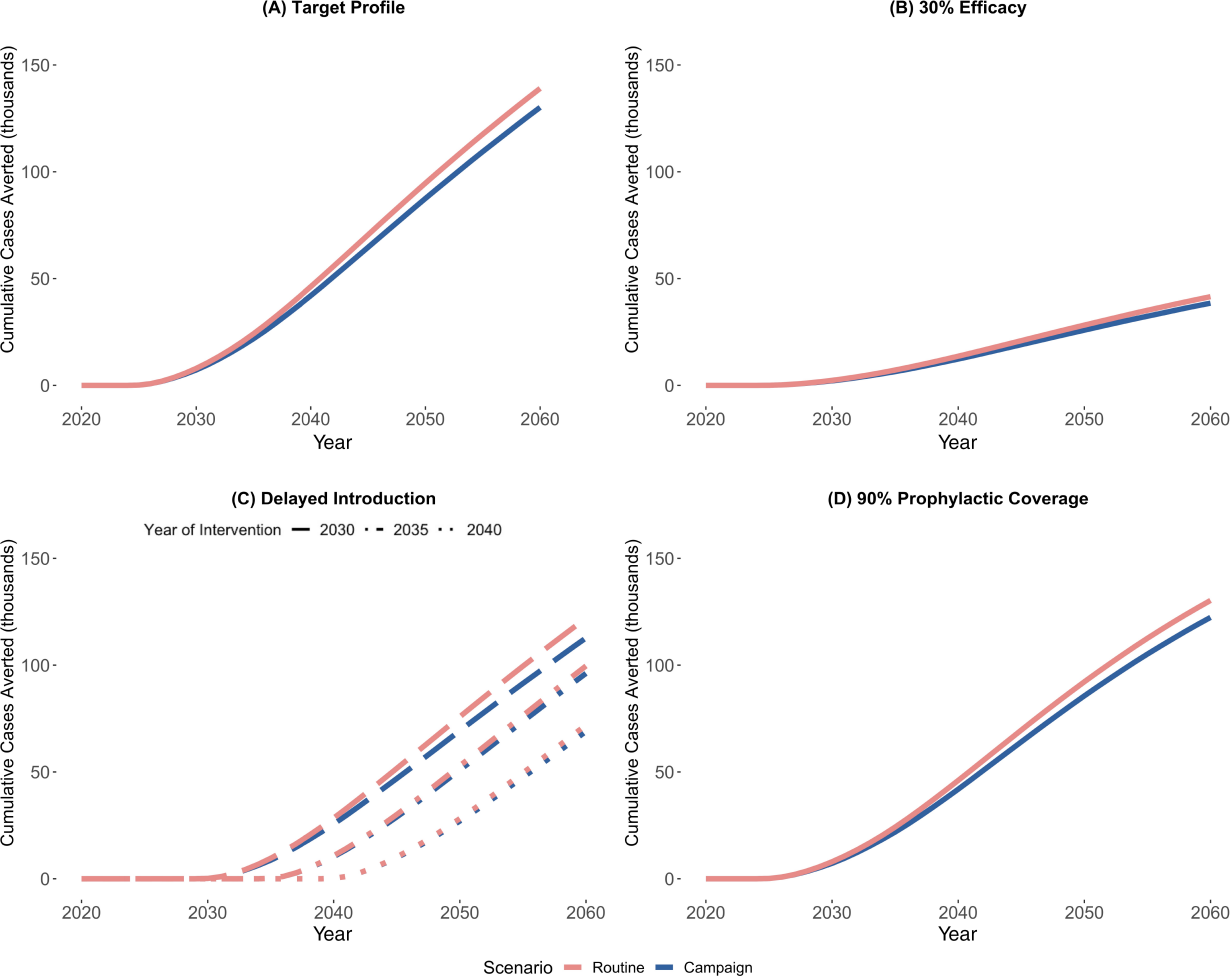
Cumulative cervical cancer cases averted through single‐contact therapeutic intervention. Each panel shows the cumulative number of cervical cases averted through a single‐contact therapeutic intervention over 40 years. Pink lines show interventions assuming routine administration at 30 years of age while blue lines show campaigns every 5 years for those 30 to 34 years of age. Panels A demonstrates the reference case (100% efficacy, introduction in 2025 and 70% prophylactic HPV vaccination coverage) and each panel represents one change from this scenario, (B) a reduction in efficacy to 30%, (C) a delayed introduction by 5, 10 or 15 years and (D) an increase in prophylactic vaccine coverage among eligible birth cohorts to 90% [Color figure can be viewed at wileyonlinelibrary.com]

When scaled up to the population‐level over 40 years in Uganda, a total of 139 050 cervical cancer cases could be averted through a routine administration approach vs 130 160 cases through a campaign approach, a 28.4% and 26.6% reduction in cancer burden relative to the status quo, respectively (Figure 3). At a lower efficacy (30% against existing HPV 16/18 infections and lesions), benefits diminished to 41 540 cases averted over 40 years through a routine approach (an 8.5% reduction). Each 5‐year delay in the introduction of a therapeutic intervention reduced the potential impact of intervention by around 20 000 cases, although this drop‐off is noticeably steeper when interventions were delayed past the year 2040, when the first birth cohorts eligible for prophylactic vaccination become eligible for the therapeutic intervention. Higher prophylactic HPV vaccine coverage (90%) starting in 2020 diminished the effectiveness of the therapeutic intervention in later years, but the effect was small (a total difference of 8740 cases relative to 70% coverage with prophylactic vaccination). Finally, removing the expanded first‐year coverage for women aged 30 to 44 years also drastically diminished effectiveness by 56 740 fewer averted cervical cancer cases over 40 years compared to the reference case (Table S1). Estimates for cumulative cases averted over 40 and 100 years for all combinations of sensitivity analysis assumptions are presented in Table S1 and online via an interactive application (https://jenniferspencer.shinyapps.io/TherapeuticHPV/).

**FIGURE 4:**
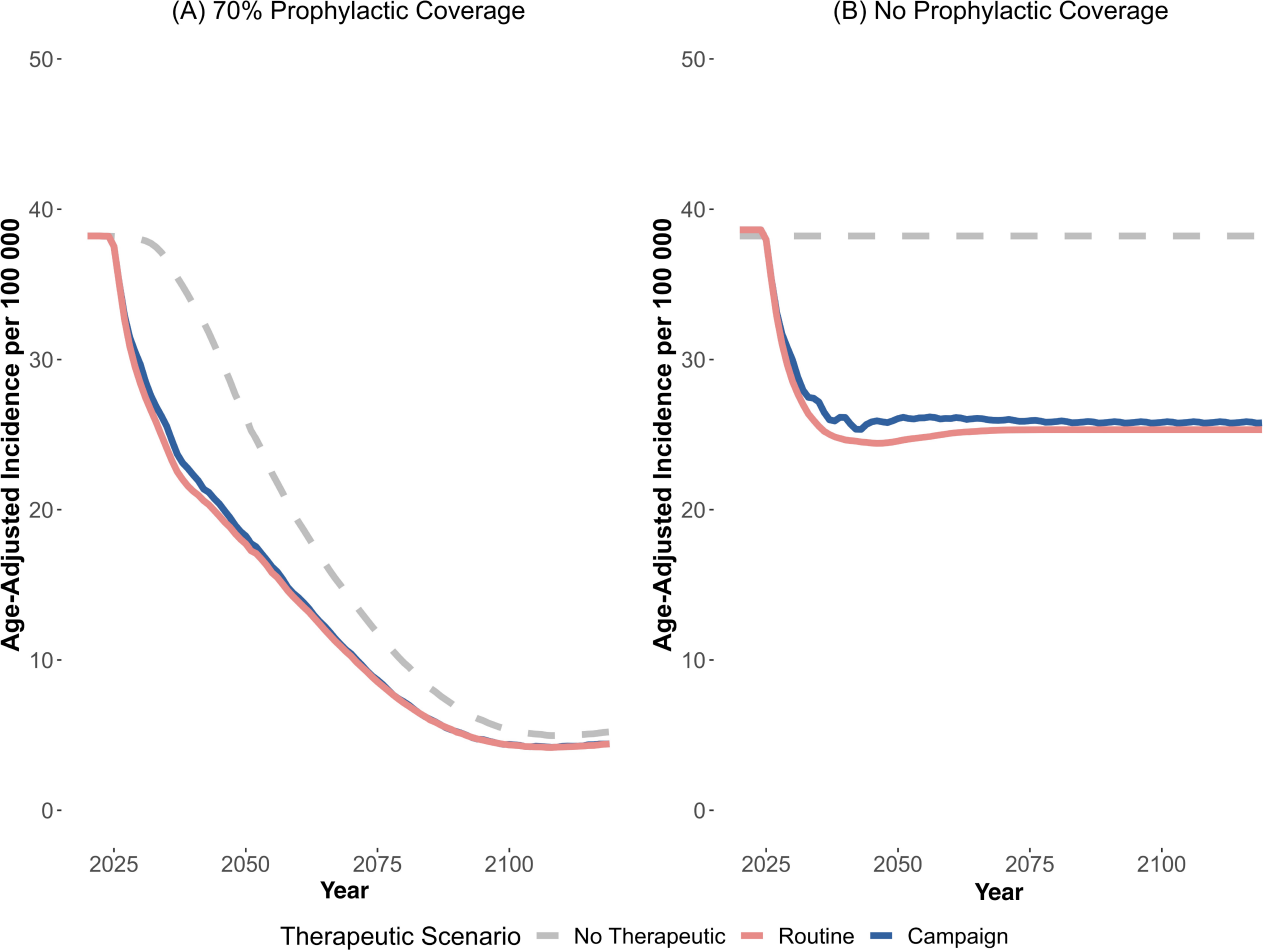
Age‐adjusted cervical cancer incidence. Panels demonstrate the age‐adjusted cancer incidence in Uganda for 100 years assuming (A) 70% prophylactic coverage among eligible cohorts or (B) no prophylactic coverage. Gray line demonstrates status quo (no therapeutic intervention, pink lines show therapeutic interventions assuming routine administration at 30 years of age with 1 year catch‐up from 30 to 45 years, while blue lines show campaigns every 5 years for those 30 to 34 years of age with 1 year catch‐up from 30 to 45 years of age [Color figure can be viewed at wileyonlinelibrary.com]

When we compared the effects of therapeutic intervention on age‐adjusted cervical cancer incidence rates over an expanded 100‐year period, in our reference case scenario, assuming 70% coverage of prophylactic HPV vaccine, cervical cancer incidence continued to decline dramatically over the next 100 years, even in the absence of therapeutic interventions (Figure 4A). While the introduction of a therapeutic intervention in 2025 resulted in a steeper initial decline in annual cases, the reduction attributable to therapeutic intervention narrowed after 2050. By 2100, routine therapeutic HPV intervention at age 30—even assuming 100% efficacy against HPV 16/18 infections and lesions—would prevent only 0.97 incident cervical cancer cases per 100 000 women annually. In contrast, in the absence of prophylactic HPV vaccination, the relative contribution of a therapeutic intervention was larger and remained high through 2100 (Figure 4B). Assuming age‐standardized cervical cancer incidence rates would otherwise remain stable in the absence of any intervention (38.2 cases per 100 000 each year), a therapeutic HPV intervention could avert 12.8 incident cervical cancer cases per 100 000 women annually by 2100, although the total burden of cervical cancer would be much larger than in the scenarios that assume prophylactic coverage.

## 4 DISCUSSION

To reduce high cervical cancer burden in Uganda, it is essential to improve current secondary prevention practices. Under highly optimistic assumptions, a single‐contact HPV therapeutic intervention was projected to avert more than 130 000 cases of cervical cancer over the next 40 years. Benefits were much lower when we assumed the therapeutic intervention was only 30% efficacious against HPV 16/18 infections and lesions (38 000 cancers averted over 40 years).

Our analysis used data on cervical cancer incidence in Uganda, but our findings have broader implications for other LMIC with high rates of cervical cancer. While Uganda has an existing HPV vaccination program, many countries with comparable cancer rates and screening use do not, and within those with vaccination programs, coverage varies widely.^7^ We present the range of possible therapeutic intervention effectiveness as background coverage of prophylactic HPV vaccination varies from 0% to 90%. In a setting without any prophylactic HPV vaccination program, therapeutic interventions could have substantially more impact, but total incidence of cervical cancer would remain high compared to settings with prophylactic vaccination alone or both prophylactic and therapeutic programs. Further, we found the timing of prophylactic vaccination roll‐out is crucial, as benefits of a therapeutic intervention fell sharply in the 20th year following the start of a prophylactic vaccine campaign with 70% coverage.

Numerous barriers to traditional approaches for secondary prevention of cervical cancer have been identified in Uganda and other LMIC, including high costs, a lack of trained providers to perform screening and necessary treatment of precancer and the need for multiple‐visit approaches that lead to high attrition.^9^, ^10^ A single‐contact therapeutic intervention could offer treatment to a large number of women without the need for multiple clinic visits or specially skilled providers. Here, we estimate an upper‐bound of potential benefit through modeling a broad intervention which offers treatment to all women without first screening for the presence of HPV infection or precancerous disease. More targeted strategies could be used to treat only women with active infections; this would reduce potential overtreatment, but also require additional resources and logistical support for upscaling screening. We note that, as of this writing, a number of novel screening, triage and treatment technologies that could facilitate a low‐cost single‐visit approach are in advanced stages of development. Additionally, new WHO guidelines for cervical cancer prevention and control recently recommended HPV DNA testing as a primary screening method over traditional cytology or VIA methods, along with providing more guidance for triage and treatment methods.^30^ Future work will need to consider the cost‐effectiveness of implementing forthcoming technologies in the context of changing prevention and screening practices.

The potential tradeoffs between ease of administration, therapeutic efficacy and timing of introduction are important to consider, especially given the current landscape of therapeutic interventions. Alternative administration modalities for HPV therapeutic interventions are possible; vaccine‐based approaches are thus far the most commonly studied, but vaginal inserts and oral medications have also been examined.^17^, ^19^, ^31^, ^32^ The few interventions that have been examined in randomized trials^20^, ^21^ demonstrated low efficacy, with an increased clearance of 15 to 20 percentage points higher than control over 6 to 9 months, even lower than our pessimistic estimate of 30%. Further, many of these products require multiple visits, which reduces potential efficacy in a real‐world setting due to potential loss to follow‐up among individuals for whom multiple clinic visits would be burdensome. Additional products are in development, but many are still in early phases, with very few having reached Phase II or Phase III clinical trials.^17^, ^18^, ^33^ Finally, we note that it takes on average 4 to 7 years after an initial submission to a regulatory agency in a high‐income country for new vaccines to be approved in sub‐Saharan Africa,^34^ and while efforts are being made to reduce this delay—it will still likely play a significant role in the timing of a potential intervention.

We note several limitations of our analysis. First, we assumed a constant therapeutic efficacy across HPV 16 and 18 infections and assumed therapeutic‐induced clearance rates to be the same regardless of infection duration and presence or severity of lesion. We did not assume clearance of any other high‐risk HPV types. Second, in our reference case we used highly favorable assumptions regarding efficacy, achievable coverage and availability of a therapeutic intervention. While we varied efficacy and timing of introduction in sensitivity analysis, other aspects of the hypothetical intervention we considered remained idealized—such as achieving immediate 70% uptake of an HPV therapeutic across all target cohorts. While our optimistic case reflects a potential upper‐bound of effects, our conservative estimates using lower efficacy and delayed introduction may still be an optimistic reflection of the low‐end estimates. Furthermore, our analysis did not account for any downsides of a therapeutic intervention, such as potential overtreatment or side effects. Previous trials of HPV therapeutics have reported generally mild side effects localized to the injection site including pain, erythema and swelling, but also report less common and more severe systemic side effects such as fatigue, lymphadenopathy and infection.^19^, ^20^, ^21^, ^35^ The extent and severity of side effects will be an important consideration as specific treatments become available.

### 4.1 Clinical and research implications

Our study is the first to evaluate the potential impact of a therapeutic HPV intervention for women of screening age in Uganda. Our findings suggest that, in order to have a meaningful impact on cervical cancer burden in Uganda and other low‐resource settings, a single‐dose therapeutic intervention will need to have considerably higher efficacy than existing interventions, be simple to administer on a large scale (ie, to achieve high coverage) and be ready to roll‐out expediently. Decision makers will need to consider the uncertain costs and benefits of a potential therapeutic intervention alongside the value of prophylactic HPV vaccination—a highly effective intervention which is already scaling up. Given that a therapeutic intervention would target women who are not generally eligible for prophylactic vaccination in low‐resource settings, there is a narrow window of opportunity for high impact. We present this exploratory analysis to inform priority setting around cervical cancer prevention strategies.

## CONFLICT OF INTEREST

The authors declare no conflicts of interest.

## ETHICS STATEMENT

This study was exempt from review by the Institutional Review Board at Harvard University because it did not involve human subjects.

## Supporting information


**Appendix S1**: Supporting Information.

## Data Availability

The model inputs, calibration data and simulated results that support the findings of this study are available from the corresponding author upon reasonable request.
