# Supplementary material for: Potential effectiveness of a therapeutic HPV intervention campaign in Uganda
Source: Int J Cancer. 2021 Nov 24;150(5):847–55. doi: 10.1002/ijc.33867 (PMC8732308; doi:10.1002/ijc.33867)
Supplement: Supplementary file 1 — Appendix S1: Supporting Information. [file IJC-150-847-s001.pdf]

Potential Effectiveness of a Therapeutic HPV Intervention Campaign in Uganda

Jennifer C. Spencer, PhD; Nicole G. Campos, PhD; Emily A. Burger, PhD;
Stephen Sy, MS; Jane J. Kim, PhD

Table of Contents

Supplemental Table 1: Cumulative Cervical Cancer Cases Averted Over 40 and 100 years........2

Supplemental Figure 1: Timing of Single-Cohort Analyses of HPV Therapeutic by Birth Cohort.4

Supplemental Figure 2: Timeline of Therapeutic Intervention Delivery Alternatives................5

Supplemental Table 1: Cumulative Cervical Cancer Cases Averted Over 40 and 100 years

Prophylactic Coverage			Therapeutic Efficacy		Year of Introduction				Start-Up Campaign (30-45)		Total Cervical Cancers Averted (Thousands)			
											40 Year Time Horizon		*100 Year Time Horizon*	
**70%**	**90%**	**0%**	**100%**	**30%**	**2025**	**2030**	**2035**	**2040**	**Yes**	**No**	**Routine**	**Campaign**	**Routine**	**Campaign**
X			X		X				X		139.0	130.2	290.8	284.8
X			X		X					X	82.3	91.9	231.4	245.0
X			X			X			X		121.9	112.8	275.9	268.8
X			X			X				X	57.3	68.7	203.8	220.2
X			X				X		X		99.6	96.1	257.2	256.2
X			X				X			X	30.0	42.8	170.5	190.5
X			X					X	X		72.0	69.0	232.8	231.5
X			X					X		X	17.2	22.1	152.4	163.4
X				X	X				X		41.5	38.5	87.6	83.8
X				X	X					X	24.7	27.1	70.0	72.0
X				X		X			X		36.5	33.4	83.2	79.1
X				X		X				X	17.3	20.2	61.9	64.7
X				X			X		X		28.5	29.8	77.6	75.4
X				X			X			X	9.2	12.6	52.0	55.9
X				X				X	X		21.6	20.6	70.4	68.2
X				X				X		X	5.3	6.5	46.6	48.0
	X		X		X				X		130.3	122.4	208.8	200.6
	X		X		X					X	73.6	84.1	149.4	160.8
	X		X			X			X		113.1	104.9	193.7	184.5
	X		X			X				X	48.6	60.8	121.8	136.0
	X		X				X		X		90.4	88.0	174.5	171.4

Color-Coding	
**40 Years**	**100 Years**
<50k	<100k
>50k, <100k	>100k, <1m
>100k	>1m

X		X				X			X	21.7	35.1	89.0	106.0
X		X					X	X		62.6	60.6	149.2	146.0
X		X					X		X	11.5	14.9	75.0	80.3
X			X	X				X		39.0	36.1	62.5	58.8
X			X	X					X	22.1	24.8	45.0	47.0
X			X		X			X		33.9	31.0	58.1	54.1
X			X		X				X	14.7	17.9	36.9	39.7
X			X			X		X		27.1	26.0	52.4	50.2
X			X			X			X	6.7	10.3	27.1	31.0
X			X				X	X		18.9	18.1	44.9	42.8
X			X				X		X	4.4	3.6	22.9	23.3
	X	X		X				X		201.2	185.9	1,216.3	1,221.3
	X	X		X					X	138.6	147.2	1,150.8	1,181.3
	X	X			X			X		183.9	168.2	1,201.3	1,205.1
	X	X			X				X	117.6	123.3	1,127.5	1,155.8
	X	X				X		X		161.7	146.0	1,183.2	1,185.6
	X	X				X			X	89.2	96.4	1,092.6	1,124.9
	X	X					X	X		133.0	118.1	1,160.9	1,161.5
	X	X					X		X	60.3	67.7	1,051.1	1,087.2
	X		X	X				X		59.8	55.3	365.4	364.9
	X		X	X					X	41.1	46.9	345.8	356.3
	X		X		X			X		54.6	50.0	306.8	360.1
	X		X		X				X	34.8	36.6	338.8	345.3
	X		X			X		X		47.8	43.2	355.3	354.2
	X		X			X			X	26.3	28.5	328.3	336.1
	X		X				X	X		39.2	35.0	348.5	347.0
	X		X				X		X	17.7	20.0	315.7	324.8

Supplemental Figure 1: Timing of Single-Cohort Analyses of HPV Therapeutic by Birth Cohort

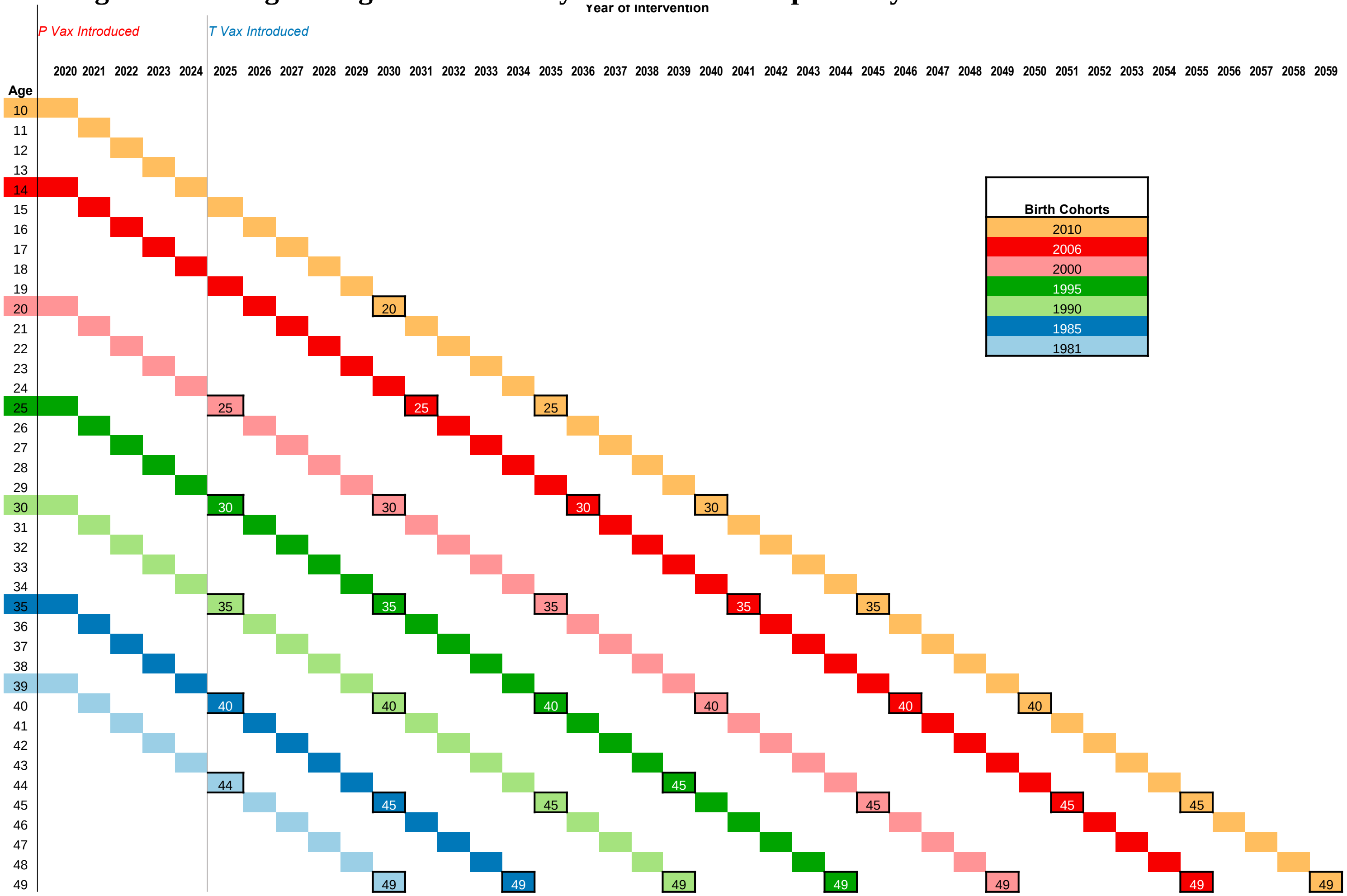

Legend. Pictured are the 7 birth cohorts included in single-cohort analyses. For each cohort, age in year 2020 is provided, followed by ages at each modeled intervention scenario in squares. For example, the 2010 birth cohort was ten years of age in 2020 when prophylactic vaccination was introduced and for this birth cohort, we compare no therapeutic intervention with six separate one-time intervention scenarios, delivered at age 25, 30, 35, 40, 45, or 49.

Supplemental Figure 2: Timeline of Therapeutic Intervention Delivery Alternatives

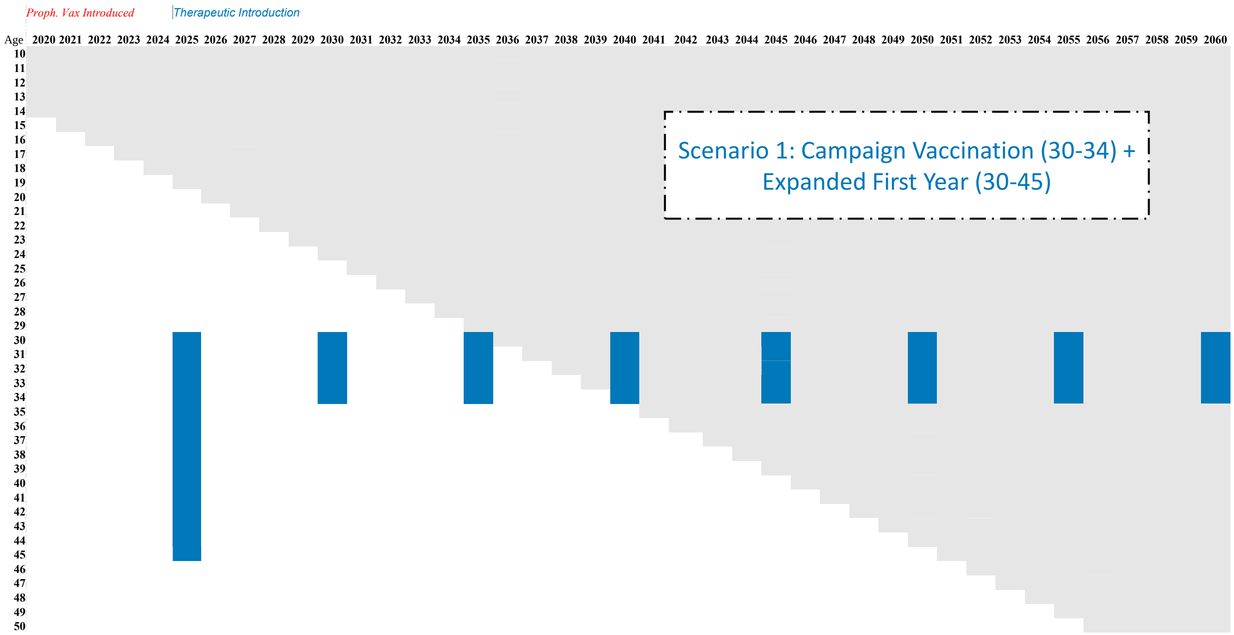

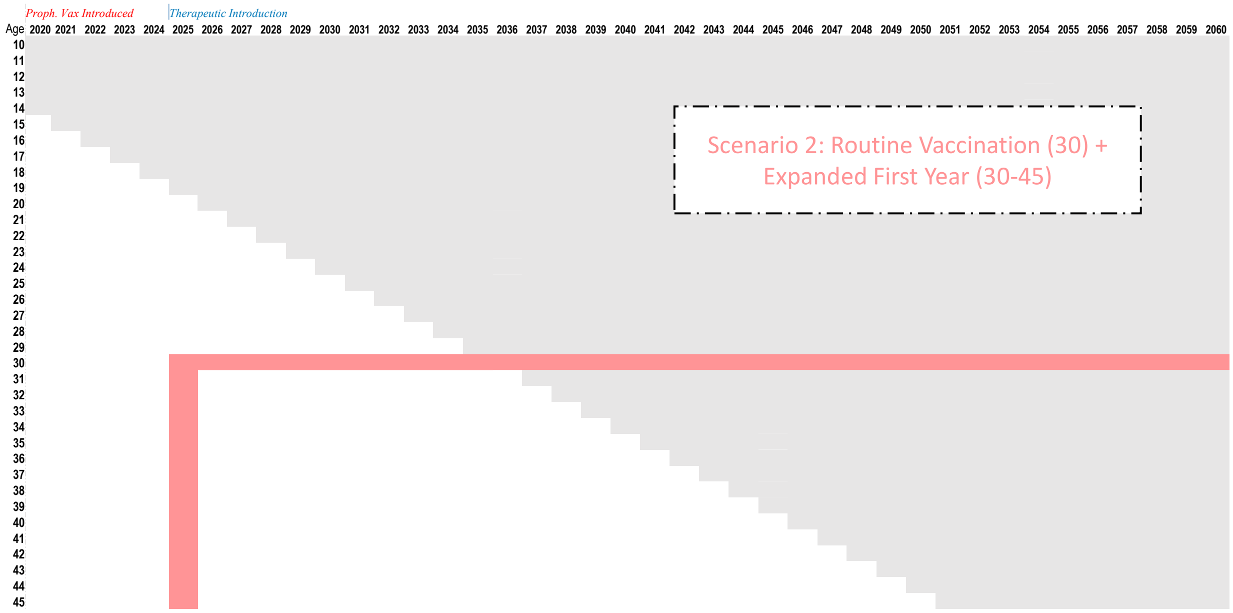

Legend. Pictured are comparative therapeutic strategies used starting in 2025 and continuing through 2060. Each row represents an age group and each column represents a year. Gray shading represents age groups potentially covered by prophylactic HPV vaccine beginning with 9-14-year-olds in 2020. The green and pink square represent age groups targeted for therapeutic intervention by year.
